# Is Component-Specific Antibody Testing Sufficient to Replace the Oral Food Challenge in the Diagnostics of Peanut-Sensitized Children? A Proof-of-Concept Study

**DOI:** 10.3390/ijms25137415

**Published:** 2024-07-06

**Authors:** Klementyna Łyżwa, Klaudia Prasek, Anna Krupa-Łaska, Joanna Zielińska, Alicja Krejner-Bienias, Magdalena Chojnowska-Wójtowicz, Wioletta Zagórska, Marek Kulus, Adam Grzela, Tomasz Grzela, Katarzyna Grzela

**Affiliations:** 1 Department of Paediatric Pulmonology and Allergology, Medical University of Warsaw, Zwirki i Wigury 63A, 05-092 Warsaw, Poland; 2 Startmed Medical Center, Partyzantow 23, 05-092 Lomianki, Poland; 3 Faculty of Mathematics, Informatics, and Mechanics, University of Warsaw, Banacha 2, 02-097 Warsaw, Poland; 4 Biostructure Research Center, Medical University of Warsaw, Chalubinskiego 5, 02-004 Warsaw, Poland

**Keywords:** adrenaline, anaphylaxis, component-specific antibody, eliciting dose, oral food challenge, peanut allergy, skin prick test

## Abstract

(1) Peanut allergy is associated with high risk of anaphylaxis which could be prevented by oral immunotherapy. Patients eligible for immunotherapy are selected on the basis of a food challenge, although currently the assessment of antibodies against main peanut molecules (Ara h 1, 2, 3 and 6) is thought to be another option. (2) The current study assessed the relationship between the mentioned antibodies, challenge outcomes, skin tests and some other parameters in peanut-sensitized children. It involved 74 children, divided into two groups, based on their response to a food challenge. (3) Both groups differed in results of skin tests, levels of component-specific antibodies and peanut exposure history. The antibody levels were then used to calculate thresholds for prediction of challenge results or symptom severity. While the antibody-based challenge prediction revealed statistical significance, it failed in cases of severe symptoms. Furthermore, no significant correlation was observed between antibody levels, symptom-eliciting doses and the risk of severe anaphylaxis. Although in some patients it could result from interference with IgG4, the latter would not be a universal explanation of this phenomenon. (4) Despite some limitations, antibody-based screening may be an alternative to the food challenge, although its clinical relevance still requires further studies.

## 1. Introduction

Peanut allergy is diagnosed in 2% of children; however, in many cases it may also persist into adulthood [1,2]. The broad distribution of peanut allergens, including those contaminating industrially processed food [3], results in significant risk of allergen exposure, which may further develop into life-threatening anaphylaxis [4].

Besides strict avoidance of peanut-containing food, peanut-specific oral immunotherapy has recently become another way to prevent severe allergic reactions, even following unintended allergen exposure [4]. However, this rapidly advancing approach requires cautious identification of patients eligible for immunotherapy, as well as determination of the initial dose of peanut protein, which could safely be used for the procedure [5].

Elevated levels of allergen-specific immunoglobulin E (IgE), positive results of skin prick tests (SPT) or basophil activation tests are commonly used to assess patient sensitization to peanut allergens [5,6]. Positive results do not invariably translate into clinical allergy manifestation.

Nevertheless, these tools do not confirm a diagnosis of allergy sensu stricto, i.e., understood as the appearance of clinical symptoms after allergen-containing food ingestion.

It is crucial to distinguish between sensitization and allergy concepts to differentiate patients who exhibit only laboratory test changes from those who require avoidance of a specific allergen due to the potential risk of undesirable symptoms. In a cohort of Polish children referred to the allergology department for diagnostic evaluation of food allergies, specific antibodies against peanut allergens were detected in 55% of individuals [7]. Given this finding and the increasing prevalence of peanut allergy among the European population, there is a pressing need for modern methods to assess the clinical significance of laboratory results.

Although a patient history of anaphylactic reactions may serve as a valuable indicator for predicting allergy to peanut proteins, it may not necessarily reflect the current sensitization state, particularly if the reaction occurred in the distant past. Moreover, some patients may present an unclear history of peanut ingestion.

Recent studies have evaluated three primary approaches for peanut immunotherapy: oral immunotherapy, sublingual immunotherapy and epicutaneous immunotherapy. The European Academy of Allergy and Clinical Immunology (EAACI) recommends allergen immunotherapy for patients who have evidence of an IgE-mediated food allergy and for whom avoidance strategies are ineffective, undesirable or significantly impair quality of life. Given that immunotherapy can be associated with a wide range of adverse effects, it is important to include children who can report side effects. Therefore, EAACI recommends qualifying patients who are over 4–5 years old. Additionally, patients and their families should demonstrate strong motivation for the procedure. The personnel conducting the therapy should have experience in managing anaphylactic reactions and have access to all necessary equipment [5].

The current method to verify a diagnosis of allergy to peanut proteins and to enroll patients in the peanut immunotherapy is the oral food challenge (OFC), specifically double-blind OFC [5,8]. Furthermore, the eliciting dose in OFC determines the initial dose used for immunotherapy.

On the other hand, the OFC procedure is associated with several practical challenges. Firstly, OFC itself carries a significant risk of serious complications, including anaphylactic shock. Therefore, this procedure requires the involvement of experienced medical professionals. Although usually it is associated with some mild discomfort, sometimes it may induce severe anxiety in patients. Last but not least, it is also a time-consuming process [8].

Recent advances in translational research focusing on the molecular composition of peanut allergens has made it possible to detect and precisely identify antibodies directed against particular allergen components [9]. The component-resolved diagnostics (CRD) platform, which was developed based on these discoveries, better discriminates between true peanut allergy and possible cross-reactive sensitizations, thereby enhancing diagnostic accuracy and potentially offering new solutions for personalized treatment strategies [10,11,12].

Among eighteen peanut proteins identified so far, three (Ara h 1, 2 and 3) have been recognized as the most important molecular indicators of sensitization. Together with two other proteins (Ara h 6 and 7), they are considered clinically relevant primary sensitization markers, which moreover belong to the most anaphylactogenic thermostable storage proteins [11]. Hence, the abovementioned component-specific antibodies (CSA), detected using CRD tools, could be considered as possible predictors of positive reaction in OFC or even of the risk of anaphylaxis [11,12,13]. Presumably, such CRD-based patient pre-selection would benefit from the reduction of the risk of anaphylactic reaction during OFC and in the course of immunotherapy. Moreover, one can speculate that CRD could replace other diagnostic methods, including skin prick test and/or OFC in the qualification of patients for oral immunotherapy.

Therefore, the aim of our study was to assess the possible relationship between serum levels of component-specific antibodies and the results of oral food challenge, skin prick test and other selected clinical parameters in a group of children sensitized to peanut and undergoing qualification for oral immunotherapy.

## 2. Results

A group of 74 children, 43 male and 31 female, was recruited according to the inclusion criteria. Approximately half of all patients had a previous history of peanut ingestion. The majority of children suffered from other atopic disease, including allergic rhinitis, other food allergies, allergies to other nuts (walnut, hazelnut, cashew) and to milk and egg, atopic dermatitis or asthma. Only 8% of patients had no history of other atopic diseases. The main details of baseline clinical characteristics, with results of skin prick tests and peanut-focused CDR screening, are summarized in Table 1.

All patients who qualified for the study underwent an open-label OFC with peanut flour. A positive reaction to the challenge (OFC+) of varying intensity was observed in 48 patients (64.9% of the whole group). The majority (83.3%) of OFC+ children presented symptoms concerning the gastro-intestinal tract—abdominal pain, nausea, vomiting and/or itching or swelling in the oral cavity. More than half (56.2%) of the OFC+ group revealed skin symptoms; there was nearly the same occurrence rate (52.1%) for nasal symptoms—mucosal swelling and rhinorrhea. Respiratory tract symptoms, mainly wheezing and/or stridor, occurred in 18.7% of patients. Symptoms concerning the circulatory system, tachycardia and decrease of blood pressure, appeared in 8.3% of patients. More than one third (37.5%) of OFC+ patients presented anxiety or somnolence. Sixteen patients (33.3%) developed severe anaphylaxis symptoms which required the administration of adrenaline.

The comparison of children categorized according to their response to OFC revealed that despite some discrepancies in mean age and sex distribution, as well as main comorbidities, there was no statistically significant difference between both groups. It is noteworthy that more than half of all children from the OFC+ group but only one fourth from the negative OFC (OFC−) group had a history of previous peanut exposure and this difference was statistically significant (*p* = 0.01).

When analyzed based on mean diameters of SPT (for both extract and flour) and mean serum levels of CSA for Ara h 1, 2, 3 and 6, all the aforementioned differed significantly between both groups (*p* < 0.001). These differences were a basis to hypothesize that for each of the aforementioned parameters there is a specific threshold which could be used to predict the patients’ further response to OFC.

**Figure 1:**
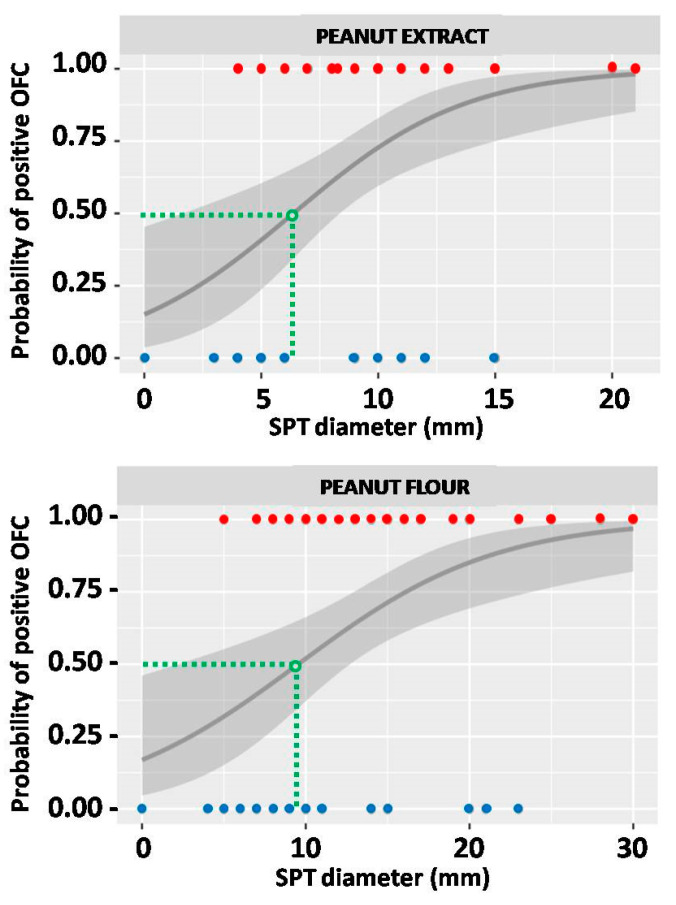
The probability of oral food challenge (OFC) outcome in relation to the result of skin prick test (SPT) with peanut extract (**upper graph**) and peanut flour (**lower graph**). Red dots represent SPT results of individuals from the OFC+ group, and blue dots those from the OFC− group. Green dashed lines indicate the SPT thresholds (expressed in millimeters), which correspond to 50% probability of positive OFC. The grey line represents probability curve, whereas grey shading corresponds to the 95% confidence intervals.

The likelihood of a positive response to the peanut OFC was assessed in relation to the diameter of the skin wheal which was formed following SPT with the use of either commercial peanut extract or peanut flour. Using the logistic regression we have estimated the wheal diameter threshold for the 50% probability of positive OFC outcome as 6.4 mm for SPT with peanut extract and 9.5 mm for peanut flour, at *p* = 0.002 for both tests (Figure 1).

**Figure 2:**
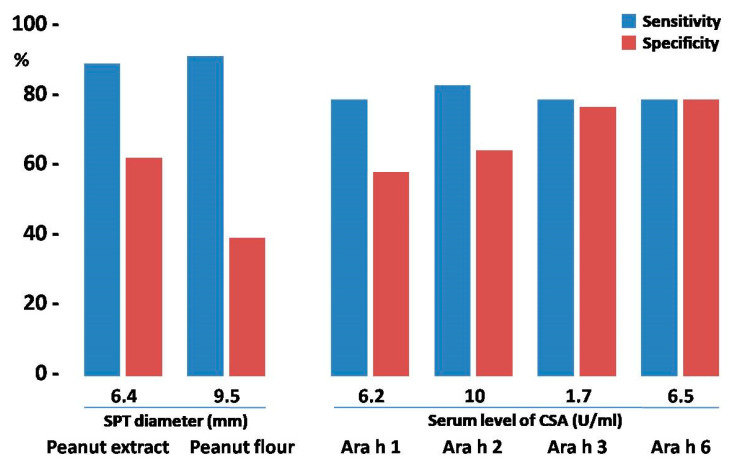
The comparison of testing efficacy of skin prick tests (SPTs) and component-specific antibodies (CSAs) with their respective thresholds. Blue bars represent the sensitivity of the test; red bars correspond to its specificity.

The abovementioned threshold for SPT with peanut extract provided a sensitivity of OFC outcome prediction at the level of 87.5%, with a specificity of 61.5%. The calculated threshold for peanut flour gave similar sensitivity (89.6%), but much lower specificity (38.5%) of such a prediction (Figure 2).

**Figure 3:**
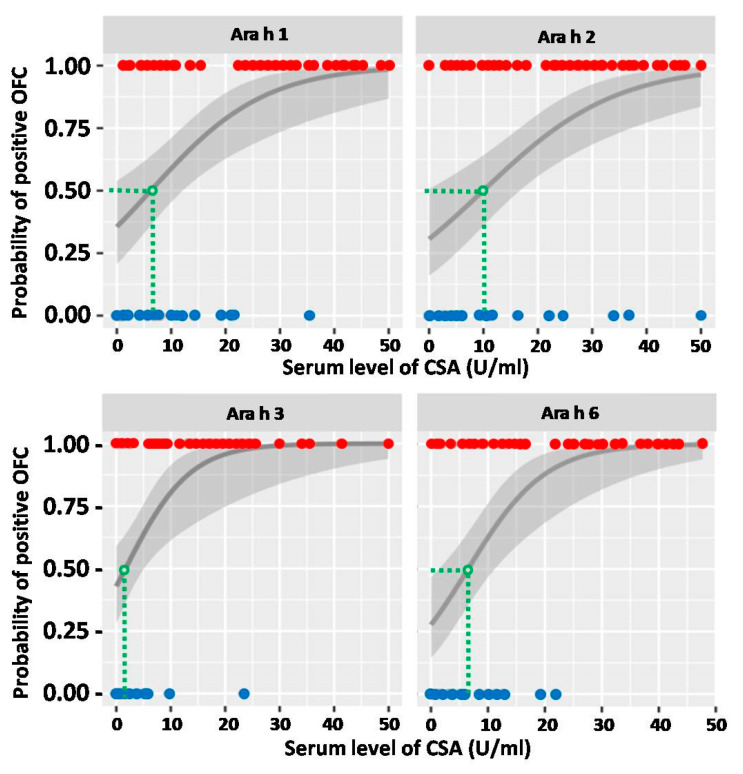
The probability of oral food challenge (OFC) outcome in relation to the serum level of respective component-specific antibody (CSA). Red dots represent CSA levels of individuals from the OFC+ group, and blue dots those from the OFC− group. Green dashed lines indicate the CSA thresholds (expressed in U/mL), which correspond to 50% probability of positive OFC. The grey line represents probability curve, whereas grey shading corresponds to the 95% confidence intervals.

The association between the OFC outcome and the levels of IgE specific to selected peanut components, Ara h 1, 2, 3 and 6, was analyzed using the same model as that for SPT. Respective serum concentration thresholds for each CSA were calculated for a 50% probability of a positive OFC outcome and the results are shown in Figure 3. Significantly, in the case of CSA of Ara h 3, the calculated threshold was as low as 1.7 U/mL. For all calculations the *p* values were below 0.005.

The calculation of sensitivity and specificity for the abovementioned CSA thresholds revealed that predictive values of CSA serum levels possessed slightly lower sensitivity (77.1–81.3%) when compared to SPT. However, in regard to specificity, it was similar (57.7–61.5%), or even better, especially for Ara h 3- and Ara h 6-specific antibodies (73.1% and 76.9%, respectively) (Figure 2).

**Figure 4:**
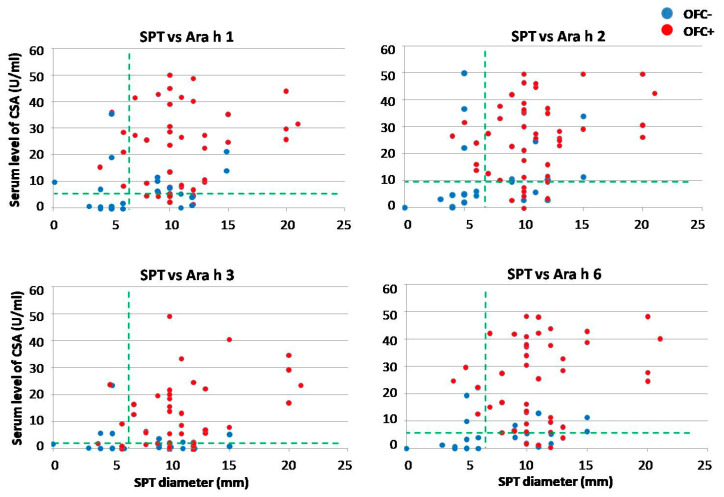
The comparison of threshold for skin prick test (SPT) in combination with those for various component-specific antibodies (CSAs). Red dots represent results of individuals from the OFC+ group, and blue dots those from the OFC− group. Horizontal green dashed lines indicate the respective CSA thresholds (expressed in U/mL), whereas the vertical lines indicate the SPT threshold (expressed in millimeters), both corresponding to 50% probability of positive oral food challenge.

**Figure 5:**
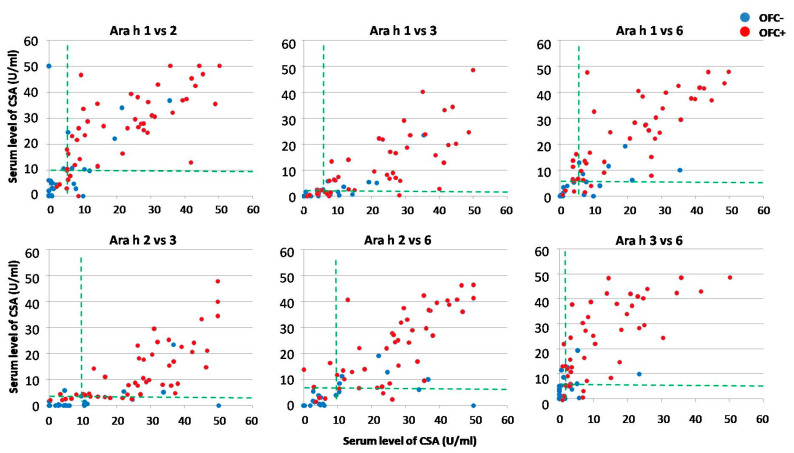
The comparison of thresholds for various combinations of component-specific antibodies (CSAs). Red dots represent results of individuals from the OFC+ group, and blue dots those from the OFC− group. Both horizontal and vertical green dashed lines indicate the respective CSA thresholds (expressed in U/mL), corresponding to 50% probability of positive oral food challenge.

In order to improve the sensitivity and specificity of OFC outcome prediction, the respective thresholds were tested in various combinations. Due to unsatisfactorily low specificity of SPT with peanut flour, only data from SPT with peanut extract were used for further analysis. Four graphs representing the combinations of SPT with each of the assessed CSAs are shown in Figure 4. Various combinations of CSAs are shown in Figure 5.

**Figure 6:**
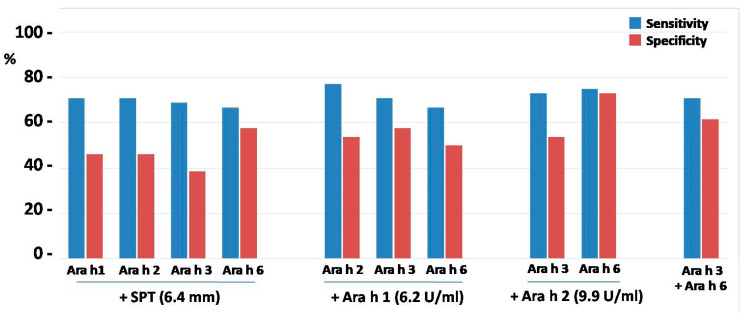
The comparison of testing efficacy of skin prick test (SPT) and component-specific antibodies (CSA) in various combinations. Blue bars represent sensitivity of the test; red bars correspond to its specificity.

The summary of sensitivity and specificity values obtained for all analyzed combinations is shown in Figure 6.

The predictive values of SPT combined with CSA levels appeared relatively low with regard to specificity of OFC outcome and were even lower as compared to CSA alone, including SPT combined with CSA for Ara h 6 (Figure 6). This was probably due to weak correlation (r value approx. 0.4) between individual results of SPT and CSA levels. However, despite much better correlations between levels of all CSAs (r > 0.7), their combinations did not improve or slightly worsened specificity as compared to single CSA, although Ara h 2 combined with Ara h 6 yielded specificity of 73.1%, similar to that of Ara h 6 alone.

Twenty eight among 48 individuals from the OFC+ group experienced peanut exposure in the past. When assessed with regards to the necessity of adrenaline use following OFC, less than half of children who required adrenaline had history of peanut exposure. In contrast to that, in the OFC+ subgroup without adrenaline, the previous peanut exposure was reported in two thirds; nevertheless, this difference was non-significant.

**Figure 7:**
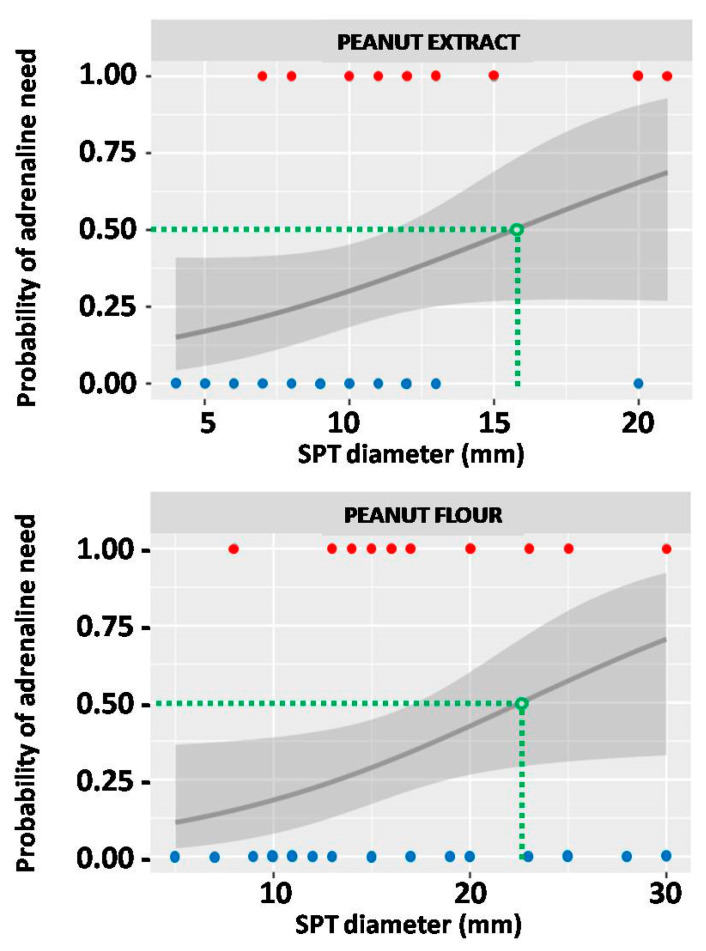
The probability of severe anaphylaxis which would require adrenaline use in relation to the result of skin prick test (SPT) with peanut extract (**upper graph**) and peanut flour (**lower graph**). Red dots represent SPT results of individuals who required adrenaline, and blue dots those who did not. Green dashed lines indicate the SPT thresholds (expressed in millimeters), which correspond to 50% probability of adrenaline use. The grey line represents probability curve, whereas grey shading corresponds to the 95% confidence intervals.

The risk of severe anaphylactic reaction, which required application of adrenaline, was assessed in relation to wheal diameter in SPT with both peanut extract and peanut flour. Using the logistic regression method, the diameter thresholds for 50% probability of anaphylaxis after OFC were calculated as 15.7 mm for peanut extract and 22.6 mm for peanut flour (*p* = 0.08 and 0.04, respectively) (Figure 7).

**Figure 8:**
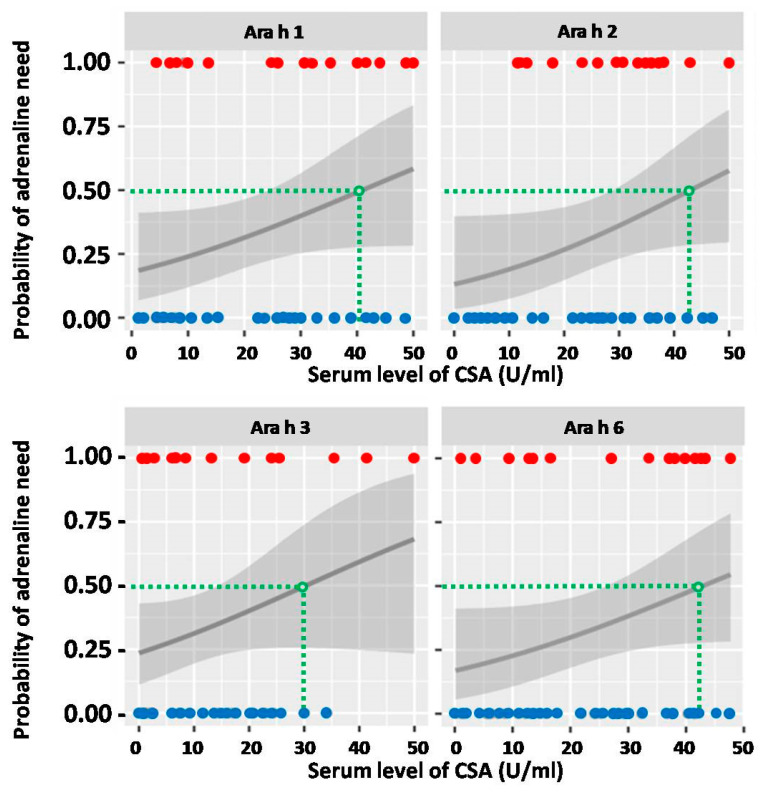
The probability of severe anaphylaxis which would require adrenaline use in relation to the serum level of respective component-specific antibodies (CSAs). Red dots represent CSA levels of individuals who required adrenaline, and blue dots those who did not. Green dashed lines indicate the CSA thresholds (expressed in U/mL), which correspond to 50% probability of adrenaline use. The grey line represents probability curve, whereas grey shading corresponds to the 95% confidence intervals.

Similar assessment was conducted for CSA levels. The thresholds calculated for 50% probability of severe anaphylaxis after OFC were as follows: 40.5 U/mL for Ara h 1; 43 U/mL for Ara h 2; 30 U/mL for Ara h 3 and 42 U/mL for Ara h 6 (Figure 8). Noticeably, none of the aforementioned calculations were considered statistically significant.

Furthermore, all calculated thresholds, when used for prediction of severe anaphylaxis risk during OFC, provided high specificity (84.4% for Ara h 2 to 93.8% for Ara h 3); on the other hand, they offered unacceptably low sensitivity (18.8% for Ara h 2 to 31.3% for Ara h 6).

When calculated for the whole group, the allergen tolerance, expressed as the cumulative eliciting dose (CED), reached the mean value 93.3 mg. While all individuals from the OFC− group received and fully tolerated the maximal cumulative dose of 144.5 mg, the mean CED in the OFC+ group was less than half of that, i.e., 67.2 ± 60.3 mg. Interestingly, the majority of OFC+ patients who did not require adrenaline application (*n* = 32) presented their symptoms rather quickly, with relatively low CED (the mean value was 45.1 ± 50.7 mg). Only 7 patients from that sub-group received CED ≥100 mg before they developed adverse reaction and accomplished OFC.

**Figure 9:**
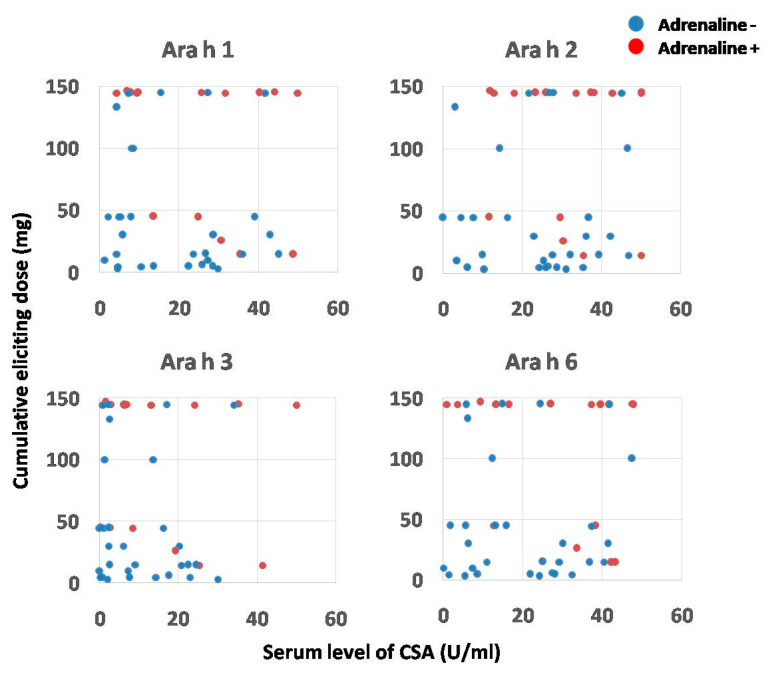
The association between serum levels of component-specific antibodies (CSAs), cumulative eliciting dose of peanut protein and the occurrence of severe anaphylaxis in patients from OFC+ group. Red dots represent individuals who required adrenaline, and blue dots those who did not.

Unexpectedly, only five of the 16 OFC+ patients who required adrenaline application developed severe anaphylactic symptoms very fast, at low dose, whereas the remaining 11 children received maximal CED (i.e., 144.5 mg) before the symptoms could fully develop. Hence, the mean CED in that subgroup was 108.7 ± 56.1 mg. The analysis of presumable associations between CED, CSA levels and risk of severe anaphylaxis with adrenaline requirement in the OFC+ group did not reveal any conclusive pattern (Figure 9).

Despite statistically significant difference in mean serum levels of peanut-specific IgG4 antibodies between OFC+ and OFC− groups (Table 1), no such difference was observed within the OFC+ group when analyzed with regard to adrenaline need.

## 3. Discussion

The oral food challenge is currently considered as a golden standard in patient qualification for peanut immunotherapy. However, the data presented in our report verify and extend some recent observations that OFC could be replaced, at least to some extent, by the screening for allergen component-specific antibodies [9,14,15,16,17]. This CSA-based approach could eliminate the risk of severe life-threatening complications, including anaphylactic shock, which could be associated with the OFC procedure. Using calculated thresholds for particular CSA levels, we could predict the result of OFC with sensitivity and specificity up to approximately 80%. The predictive value of CSA for OFC outcome, especially in the case of Ara h 3 and 6, was even better than skin prick tests, which are recommended as a first line of diagnosis for patients suspected of suffering from food allergy [6]. The SPT performed with peanut extract, when applying a calculated threshold of 6.4 mm of wheal diameter, was found to be a sensitive (up to 90%) but a relatively low-specificity (up to 60%) predictor of positive OFC. It is noteworthy that it was significantly lower than the 8 mm threshold reported in other studies [18,19,20]. Furthermore, the cut-offs for CSA, especially for Ara h 2, strongly differ between various authors [11,16]. Apart from different diagnostic platforms used for CSA screening, these discrepancies could presumably result from a lower final dose of peanut protein (max 100 mg) used in our OFC protocol, compared to max 2000 mg used by other authors [15]. Another explanation could be the higher sensitization level observed in patients recruited for our study. Significantly, in our observation the calculated thresholds for CSA matched with respective mean serum levels in the OFC− group. These, however, were much higher when compared to OFC− patients from other studies [16]. 

An attempt to combine SPT with CSAs, or use the combinations of different CSAs, did not improve the sensitivity or specificity of our tests. This was presumably due to more restrictive criteria. Accordingly, only individuals positive for both parameters were labeled “true-positive”, whereas those double-negative were considered “true-negative”. Hence, individuals, who were positive for only one parameter, were identified as “false-negative”, whereas negatives with one positive parameter were labeled “false-positive”. Since this approach increased the frequency of false-positive and false-negative results, it obviously resulted in a reduction of sensitivity and/or specificity of the test. Although the double-positive and double-negative results should be considered more convincing, even for the most effective combination, Ara h 2 with Ara h 6, the test did not exceed 80% of sensitivity and specificity.

Another benefit of using combined testing is the more accurate discrimination between real food allergy and cross-reactivity or sensitization only. Since all tested CSAs recognized various storage proteins, it is unlikely that polysensitized patients with high CSA levels will be tolerant to high doses of peanut protein. Hypothetically, the optimal combinations should involve specific IgE antibodies directed against various classes of allergen molecules, i.e., CSAs specific to peanut globulins (Ara h 1 and 3), combined with CSAs against peanut albumins (Ara h 2 and 6). In our material, such a combination (CSAs for Ara h 3 and Ara h 6) gained 70.8% sensitivity and 61.5% specificity in the prediction of OFC results, whereas the combination of CSAs directed against two peanut albumins, Ara h 2 and 6, resulted in a superior sensitivity and specificity (with 75% and 73.1%, respectively). The strong connection between co-sensitization to Ara h 2 and 6 and the severity of peanut allergy was also suggested by other authors [12].

The precise identification of diagnostic cut-off points for peanut-sensitized patients has several practical applications. Firstly, CSA screening seems to be a valuable tool to distinguish between patients who are only sensitized and are likely to tolerate a specific quantity of the allergenic food protein from those who can develop severe symptoms, including anaphylactic shock [21]. The association between serum levels of selected CSAs and the risk of severe anaphylaxis was non-significant, although some trends were observed. The lack of statistical significance was also observed with regard to the correlation between symptom severity and the eliciting dose used for OFC. Surprisingly, two thirds of patients who required adrenaline application developed symptoms relatively late, after maximal CED. In contrast, only the remaining one third experienced anaphylactic shock after low CED. Significantly, no association between individual CSA levels and CED in both sub-groups was observed.

We could not find any clear explanation for this phenomenon. Presumably, it could be due to the CSA interference with peanut-specific IgG4 antibodies [22,23]. Such antibodies could bind the same epitopes and block their interaction with IgE on mastocytes or precipitate the circulating allergen, hence decreasing its effective dose and reducing the risk of severe systemic response. This scenario could take place at least in some patients. Indeed, in serum samples of three children who required intensive adrenaline intervention after full CED we found significantly higher IgG4 levels (>4.0 µg/mL), as compared to either the whole group, the OFC+ group or its adrenaline-treated sub-group. On the other hand, in the same adrenaline-treated subgroup there were five other patients who also developed severe anaphylaxis symptoms after full CED, although their IgG4 levels were all below 0.6 µg/mL. Similar divergences with regard to possible impact of IgG4 on CED were observed in OFC+ patients, who did not require adrenaline, regardless of the fact that the mean IgG4 level in that subgroup was lower as compared to the previous one. It is plausible that instead of IgG4 alone, the CSA/IgG4 ratio would be more useful to improve the predictive value of CSA.

## 4. Materials and Methods

The study group was recruited from children referred to the Department of Pediatrics, Allergology and Pulmonology, at the University Hospital of the Medical University of Warsaw, between 2020 and 2023, for a routine evaluation of suspected peanut allergy. The inclusion criteria encompassed individuals between 4 and 18 years old, with a history of previous peanut reactions, available serum tests to confirm peanut sensitization and positive results of SPT with peanut allergens. All children were subjected to open-label OFC with peanut flour. Based on patients’ response to the food challenge, they were allocated into two groups, positive (OFC+) or negative (OFC−), and their data were subjected to further assessments and comparisons between groups. All procedures were performed according to the Declaration of Helsinki; all children as well as their parents or legal guardians gave written informed consent to participate in the study, which was approved by the Ethics Committee at the Medical University of Warsaw (KB/126/2019).

The skin prick tests were performed using both peanut extract in concentration 3 mg/mL (Diater Pharmaceutical Laboratory, Madrid, Spain) and native defatted peanut flour suspended in saline (Charrak Nutrition GmbH, Ramstein-Miesenbach, Germany). The allergens were applied on the ventral surface of the forearm skin using sterile lancets. The positive control with histamine and the negative one with saline were also included. SPT results were evaluated after 15 min with a positive response defined as a wheal with diameter of ≥3 mm.

The serum samples were tested using ALEX2–Allergy Explorer (ALEX^®^; MacroArray Diagnostics, Wien, Austria). This multiparametric assay enabled the simultaneous screening of specific IgE antibodies directed against a wide range of potential allergens, including peanut components, particularly Ara h 1, 2, 3 and 6. Sensitization was defined as the IgE serum concentration exceeding 0.3 U/mL.

An open-label oral food challenge was performed according to PRACTALL guidelines [13] in a clinical setting allowing any adverse events to be managed by experienced medical staff. Prior to the OFC all patients avoided peanuts in their diet. The OFC was performed at least 6 months after the last known exposure to peanuts. However, some participants did not exclude the foods labeled as “may contain peanut contaminants”. Children with evidence of current infection or exacerbation of atopic diseases were not qualified for the challenge.

Defatted peanut flour containing approximately 50% of whole peanut protein was suspended in fruit mousse. The portions with consecutively increasing protein amounts, 1.5 mg, 3 mg, 10 mg, 30 mg and 100 mg, suspended in approximately 1 tablespoon of fruit mousse, were administered at 30 min intervals. The result of OFC was considered positive when any of objective symptoms like urticaria, erythema, abdominal pain, vomiting, persistent rhinorrhea, stridor, wheezing or decrease of blood pressure occurred. For patients with subjective symptoms the challenge was assessed as positive, if symptoms lasted more than 40 min, or at least 3 subjective symptoms occurred simultaneously.

The eliciting dose was defined as a single dose, after which the first adverse reaction occurred. The cumulative eliciting dose was determined as the total dose of peanut protein, leading to completion of OFC.

The necessity of adrenaline administration was considered in patients, who developed severe objective symptoms at least in two organs/systems, e.g., urticaria with stridor or wheezing, rhinorrhea with abdominal pain and vomiting, etc., or presented anaphylactic shock with decrease in blood pressure.

Patients without any symptoms following OFC were observed in the clinic up to 4 h after the challenge. If no symptoms emerged during this follow-up period, the result of the procedure was considered negative.

The distribution of the analyzed variables was tested using the Shapiro–Wilk test, rejecting the null-hypothesis that the population is normally distributed at a p-value of below 0.05. The patients’ clinical characteristics, including their age, results of SPT, serum concentrations of component-specific antibodies, as well as eliciting doses applied during OFC were analyzed using descriptive statistics with the calculation of the arithmetic mean, median and standard deviations. Then, they were compared between groups using an unpaired Student’s *t*-test.

The other parameters including sex distribution, medical history (particularly focused on concomitant allergies and previous peanut exposures), the occurrence of allergy symptoms following OFC and need for adrenaline application were shown as respective ratios or percentages. Values representing the ordinal scale were compared among groups using the Mann–Whitney U test. Variables of the nominal scale were compared between groups using Pearson’s chi-squared test. The observed differences were considered statistically significant at *p* < 0.05.

The association between results of SPT or serum concentrations of peanut CSA and OFC outcome were analyzed in the whole group using logistic regression. This method enabled the calculation of respective thresholds, either wheel diameter (for SPT) or serum concentrations (for specific antibodies) corresponding to some arbitrary pre-defined probability (e.g., 50%) of positive OFC (OFC+). These settings were used alone or in combinations for the calculation of sensitivity and specificity of such a diagnostic tool.

A similar assessment was conducted for the possible association between results of SPT or serum concentrations of CSA and the need for adrenaline use in patients from the OFC+ group. The calculations were conducted to determine respective thresholds gaining the 50% probability of adrenaline requirement.

All calculations were conducted using the MedCalc Software 18.11v (www.medcalc.org, accessed on January–April 2024).

## 5. Conclusions

Based on current knowledge, the answer to the question posed in the title appears to be negative. Component-resolved diagnostics cannot serve as a substitute for the oral food challenge, but it may aid in selecting patients who have a clear indication for OFC and those who are eligible for oral immunotherapy. It can also provide risk reduction and improved diagnostics of peanut allergic patients. Establishing standardized thresholds for CSA levels will enhance the consistency and reliability of peanut allergy diagnoses across different medical centers and studies.

In contrast to OFC, component-resolved diagnostics does not enable a direct determination of the initial allergen dose, which could safely be used for immunotherapy. This main limitation of CSA-based predictive models is further extended by their weak effectiveness with respect to determining the risk of severe anaphylactic reactions. While some authors have suggested there is a strong correlation of the latter with the history of previous peanut exposure, our data did not confirm that observation. Despite the aforementioned limitations, CSA-based screening seems to be a promising alternative to the oral food challenge, although its introduction to real-life clinical use still requires further studies. Conducting research with larger patient cohorts, further studies into the CSA/IgG4 ratio and expanding the range of allergen components analyzed in CSA screening may be the most important directions in further research. By addressing these areas, future studies can build on our findings to refine peanut allergy diagnostics, improving patient care and safety.

## Figures and Tables

**Table 1:** The clinical characteristics of the study group.

Parameter/Variable	Whole Group (n = 74)	OFC+ (n = 48)	OFC− (n = 26)
Demographics			
Mean age (median); ±SD	6.9 (6); ±3.0	7.2 (7); ±2.8	6.3 (5); ±3.2
Male (%)	43 (58%)	24 (50%)	19 (73%)
Female (%)	31 (42%)	24 (50%)	7 (27%)
Comorbidities (%)	68 (92%)	47 (97.9%)	21 (80.8%)
Other food allergies (%)	48 (65%)	30 (62.5%)	18 (69.2%)
Allergic rhinitis (%)	53 (71.6%)	37 (77%)	16 (61.5%)
Asthma (%)	32 (43.2%)	22 (45.8%)	10 (38.5%)
Atopic dermatitis (%)	33 (44.6%)	21 (43.75%)	12 (46.1%)
History of peanut exposure (%)	35 (47.2%)	28 (58.3%) *	7 (26.9%) *
Allergy tests			
Mean SPT–flour (median); ±SD [mm]	14.4 (15.0); ±6.3	16.1 (15.0); ±6.0 *	11.0 (10.0); ±5.6 *
Mean SPT–extract (median); ±SD [mm]	9.7 (10.0); ±4.1	10.9 (10.0); ±3.7 *	7.4 (6.0); ±4.0 *
Mean Ara h 1 (median); ±SD [U/mL]	16.1 (9.9); ±14.8	21.3 (22.5); ±14.8 *	6.5 (2.1); ±8.7 *
Mean Ara h 2 (median); ±SD [U/mL]	20.9 (21.9); ±15.6	26.4 (26.9); ±13.9 *	10.4 (5.0); ±13.1 *
Mean Ara h 3 (median); ±SD [U/mL]	8.5 (2.6); ±11.3	12.0 (7.2); ±12.2 *	2.1 (0.2); ±4.8 *
Mean Ara h 6 (median); ±SD [U/mL]	16.5 (11.4); ±15.5	23.0 (24.2); ±15.1 *	3.9 (1.5); ±5.1 *
Mean IgG4 (median); ±SD [µg/mL]	1.68 (0.7); ±2.8	2.07 (1.0); ±3.3 *	0.95 (0.4); ±1.1 *

* Statistically significant at *p* < 0.05.

## Data Availability

The data presented in this study are available on request from the corresponding author. The original data are not publicly available due to [law restrictions to protect patient confidentiality].
